# Quantitative Regulation of Recrystallization Behavior and Grain Boundary Characteristics of Stainless Steel Ultra-Thin Strips by Electrical Pulse Treatment

**DOI:** 10.3390/ma19214450

**Published:** 2026-10-22

**Authors:** Bingchao Wang, Wanwan Fan, Jun Li, Feng Xu, Tianxiang Wang, Jinzhu Fu, Xiaoquan Han, Qiyao Zhe

**Affiliations:** 1 National Major Laboratory of Advanced Stainless Steel, Taiyuan Iron and Steel (Group) Co., Ltd., Taiyuan 030024, China; 2 College of Mechanical Engineering, Taiyuan University of Technology, Taiyuan 030024, China; 13546325484@163.com; 3 Shanxi Taigang Stainless Steel Precision Strip Co., Ltd., Taiyuan 030024, China

**Keywords:** SUS304 stainless steel strip, electric pulse treatment, recrystallization behavior, special grain boundaries, prediction model

## Abstract

**Highlights:**

- Current density, pulse frequency, duty cycle, and treatment duration exhibit distinct sensitivity windows for recrystallization evolution.
- Initial deformation-induced GND density strongly influences the recrystallization response under electrical pulse treatment.
- A semi-empirical model incorporating electrical pulse parameters and GND density accurately predicts the recrystallized fraction (R^2^ = 0.9545).
- Quantitative relationships between recrystallized fraction and HAGB/Σ3^n^ special grain boundaries are established.

**Abstract:**

To reveal the effects of electrical pulse treatment parameters and initial deformation states on the recrystallization behavior and grain boundary characteristics of SUS304 stainless steel ultra-thin strips, electrical pulse treatments were systematically conducted by varying current density (1.50–1.80 A·mm^−2^), pulse frequency (50–3000 Hz), duty cycle (5–20%), and treatment duration (5–20 s) under their respective controlled conditions. Electron backscatter diffraction (EBSD) was employed to analyze the evolution of recrystallized fraction, local misorientation, grain boundary types, and Σ3^n^ special grain boundaries. The results demonstrate that electric pulse treatment significantly promotes recovery and recrystallization of cold-rolled microstructures, while current density, pulse frequency, and duty cycle exhibit distinct sensitivity windows. Specimens with higher initial deformation levels show stronger dislocation recovery and recrystallization responses under identical electric pulse treatment conditions. Furthermore, based on the classical Johnson–Mehl–Avrami–Kolmogorov (JMAK) model, a semi-empirical prediction model for static recrystallization fraction was established by incorporating electric pulse treatment parameters and initial geometrically necessary dislocation (GND) density, achieving a coefficient of determination, R^2^, of 0.9545. Quantitative correlations between recrystallized fraction and high-angle grain boundaries (HAGBs) and low-angle grain boundaries (LAGBs) as well as Σ3^n^ special grain boundaries were further established. This work provides new insights into the quantitative control of recrystallization behavior and grain boundary evolution in ultra-thin metallic strips through electric pulse treatment.

## 1. Introduction

Stainless steel ultra-thin strips exhibit excellent mechanical strength, ductility, and corrosion resistance, making them widely used in precision manufacturing fields such as aerospace, micro-electromechanical systems, and electronic devices [1,2]. However, during the cold-rolling process of ultra-thin strips, increasing deformation leads to significant dislocation accumulation, resulting in severe work hardening and residual stress, which complicates subsequent microstructure and property regulation [3]. Although conventional annealing can reduce dislocation density and restore cold-rolled microstructures through recovery and recrystallization, it generally suffers from prolonged processing time, high energy consumption, and limited process flexibility [4,5]. In contrast, electric pulse treatment features a short processing duration, concentrated energy input, and flexible parameter controllability, which can promote dislocation motion, recovery, and recrystallization in deformed metals [6,7,8]. Compared with conventional thermal treatment, these characteristics enable rapid and tunable energy delivery and provide greater flexibility for controlling microstructural evolution. Therefore, electrical pulse treatment shows potential as a rapid and flexible approach for microstructural regulation in a broader range of metallic materials.

Previous studies have demonstrated that electrical pulse treatment-induced microstructural evolution is highly sensitive to processing parameters, and variations in current density, pulse frequency, and treatment conditions can significantly alter the dislocation state and recrystallization kinetics of metallic materials [9,10]. Dai et al. [11] reported that increasing the electrical pulse frequency could accelerate dislocation movement and significantly modify the microstructural characteristics of materials. Jeong et al. compared electrical pulse treatment with conventional thermal annealing and found that electrical current could substantially accelerate recrystallization kinetics; furthermore, the electrical pulse-assisted recrystallization process was quantitatively described based on the classical JMAK recrystallization kinetics model [12]. On the other hand, the formation of HAGBs and Σ3^n^ special grain boundaries during recrystallization is not solely determined by treatment temperature, but is also closely associated with the initial deformation state, recrystallization degree, and subsequent grain boundary migration [13]. These studies indicate that current research on electrical pulse-assisted microstructural regulation has gradually progressed from qualitative observations of dislocation and microstructural evolution toward quantitative descriptions of recrystallization kinetics and grain boundary characteristics. Therefore, microstructural evolution during electrical pulse treatment is governed by the coupled effects of electrical pulse parameters and the initial cold-rolled state [14]. Although considerable efforts have been devoted to understanding the effects of individual parameters, most previous studies have focused on individual electrical pulse parameters or specific aspects of microstructural evolution. quantitative relationships between recrystallized fraction and grain boundary characteristics under the combined effects of multiple electrical pulse parameters (current density, frequency, duty cycle, and duration) and initial stored dislocations remain insufficiently explored. In particular, a unified model describing the relationship among electrical pulse parameters, recrystallization degree, and grain boundary characteristics is still lacking.

In this work, SUS304 stainless steel ultra-thin strips with different cold-rolling reductions were selected to systematically investigate the effects of current density, pulse frequency, duty cycle, and treatment duration on static recrystallization behavior and grain boundary characteristics. Combined with kernel average misorientation (KAM) characterization, the influence of initial stored dislocations on the recrystallization response under electrical pulse treatment was analyzed. Meanwhile, conventional heat treatments under identical temperature and duration conditions were conducted for comparison. Based on the classical JMAK model, a semi-empirical predictive model coupling electrical pulse parameters with the initial microstructural state was established to quantitatively predict the recrystallized fraction. Furthermore, quantitative correlations between the recrystallized fraction and the fractions of HAGBs and Σ3^n^ special grain boundaries were established, thereby linking electrical pulse processing, initial deformation state, recrystallization behavior, and grain boundary characteristics within a unified quantitative framework.

## 2. Materials and Methods

The raw material used in this study was SUS304 stainless steel ultra-thin strip with a width of 200 mm and a thickness of 50 μm, and its chemical composition is listed in Table 1. The strips were subjected to multi-pass cold rolling at room temperature using a 20-high precision cold-rolling mill. Samples were collected after each rolling pass to obtain cold-rolled ultra-thin strips with different deformation levels. After one, three, and five rolling passes, cold-rolled samples with thicknesses of 39 μm, 29 μm, and 23 μm were obtained, corresponding to rolling reductions of 22%, 42%, and 54%, respectively.

**Figure 1:**
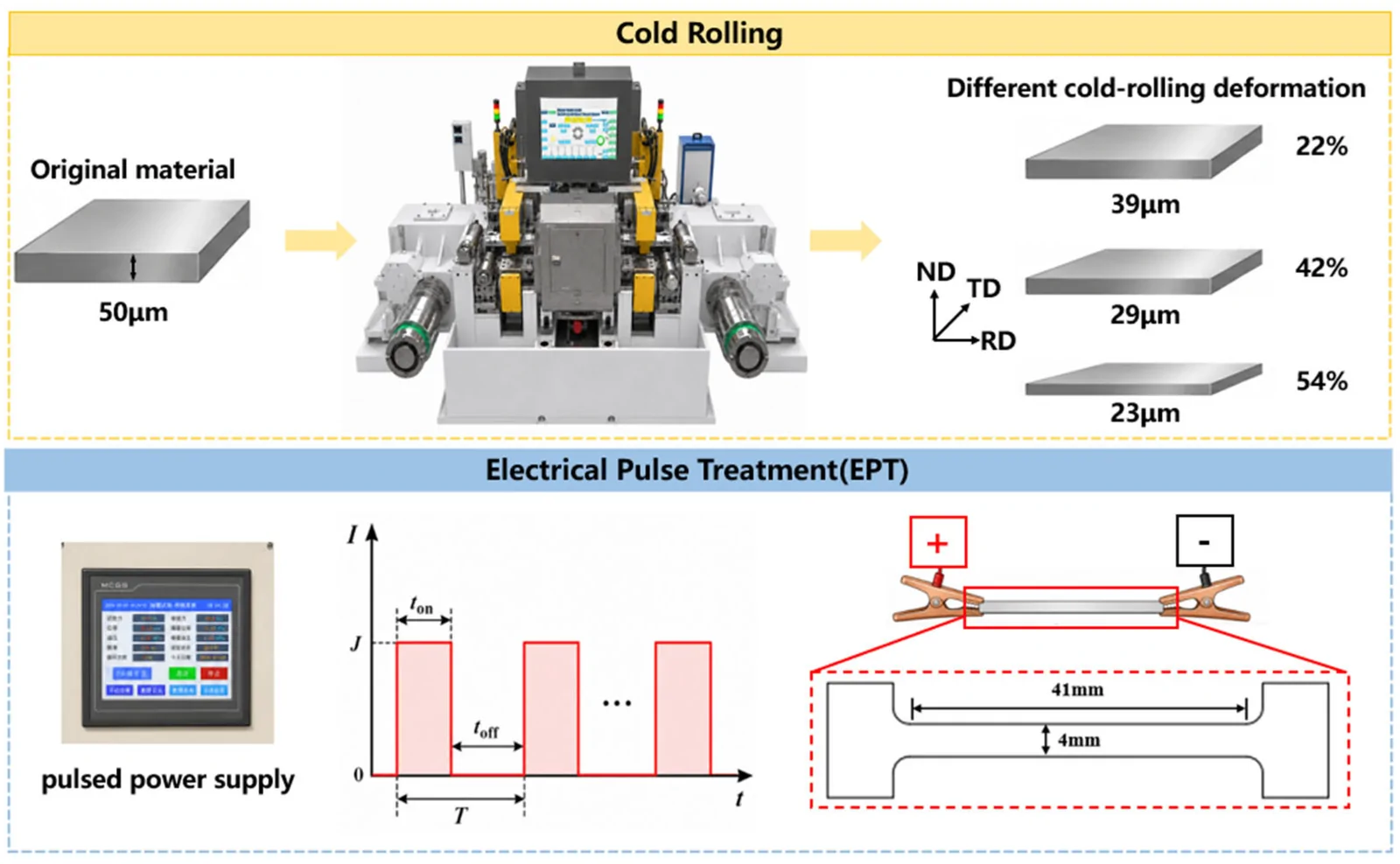
SUS304 stainless steel strip cold rolling and electric pulse treatment experimental process diagram.

A rectangular-wave DC pulse power supply (YS9000DB-30) was used to perform electrical pulse treatment experiments on one-pass cold-rolled samples under different processing conditions. The effects of electrical pulse treatment parameters, including current density (1.5, 1.55, 1.60, 1.70, and 1.80 A·mm^−2^), pulse frequency (50, 100, 1500, and 3000 Hz), duty cycle (5%, 10%, and 20%), and treatment duration (5, 10, and 20 s), on the recrystallization behavior of SUS304 stainless steel ultra-thin strips were systematically investigated. Furthermore, the cold-rolled ultra-thin strips with three different rolling reductions were treated using the selected electrical pulse treatment parameters to investigate the effect of initial deformation degree on the recrystallization response. The detailed experimental procedure is illustrated in Figure 1. During electrical pulse treatment, the specimen temperature was monitored to characterize the Joule-heating response induced by the applied current. No external closed-loop temperature control was applied; therefore, the specimen temperature evolved naturally with the electrical pulse parameters and treatment duration. The measured temperature data were used to evaluate the thermal contribution during electrical pulse treatment and to provide a basis for comparison with conventional thermal treatment.

All prepared samples were characterized by EBSD on the RD-TD plane using a JEOL-IT500 scanning electron microscope(JEOL Ltd., Tokyo, Japan). The EBSD specimens were cold-mounted and mechanically polished using diamond suspensions to remove surface scratches, followed by vibration polishing to eliminate the surface deformation layer. EBSD measurements were performed at an accelerating voltage of 20 kV, a specimen tilt angle of 70°, and a magnification of 500-fold, with a step size of 0.4 μm. The EBSD data were analyzed using Aztec Crystal software (version 2.1) to obtain the microstructural features, grain boundary distributions, recrystallization states, and local misorientation distributions of each sample.

## 3. Results and Discussion

### 3.1. Effect of Electrical Pulse Treatment Parameters on Recrystallization Behavior of SUS304 Stainless Steel Ultra-Thin Strips

#### 3.1.1. Effect of Current Density on Recrystallization

**Figure 2:**
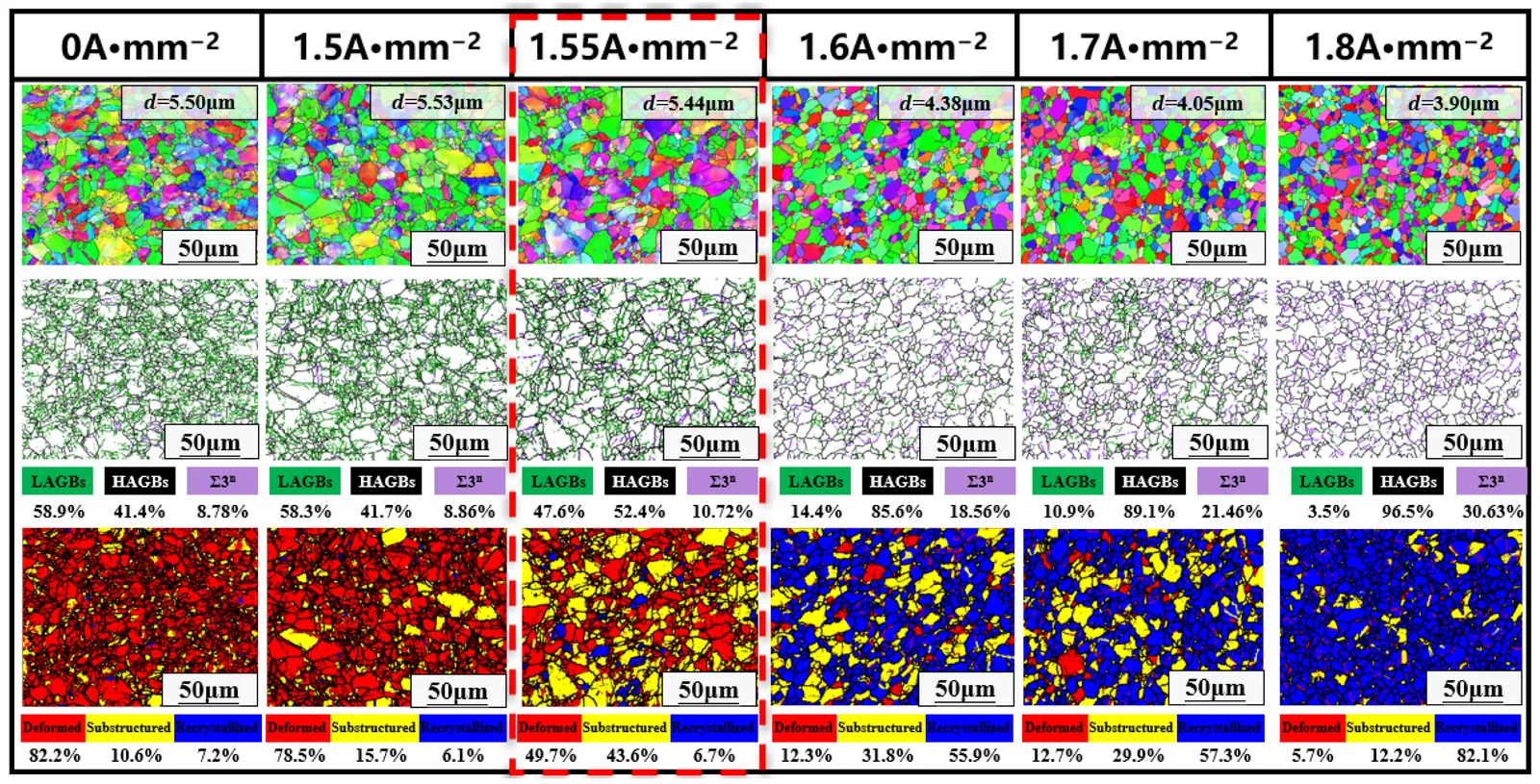
Microstructures of one-pass cold-rolled samples after electrical pulse treatment with different current densities. In the grain-boundary maps, green and black lines represent LAGBs and HAGBs, respectively, and Σ3^n^ special grain boundaries are purple lines. In the recrystallization-state maps, red, yellow, and blue represent deformed, substructured, and recrystallized regions, respectively. The same legend convention is used in the subsequent EBSD figures.

Based on a pulse frequency of 100 Hz, a duty cycle of 10%, and a treatment duration of 5 s, electrical pulse treatments with different current densities (1.50, 1.55, 1.60, 1.70, and 1.80 A·mm^−2^) were performed on one-pass cold-rolled samples, and the results are shown in Figure 2. At a current density of 1.50 A·mm^−2^, the microstructure of the electrical pulse-treated sample was similar to that of the cold-rolled state, with deformed grains accounting for 78.5%. With increasing current density, the microstructure of the ultra-thin strips gradually transformed from a highly deformed structure toward a recrystallized structure. At 1.55 A·mm^−2^, localized recovery and recrystallization occurred, accompanied by a decrease in the fraction of deformed grains to 49.7% and an increase in substructured grains to 43.6%. This behavior can be attributed to the promotion of dislocation movement by electrical pulse treatment, which reduces local orientation gradients within grains and facilitates the gradual transformation of highly deformed grains into substructured regions.

When the current density increased to 1.60 A·mm^−2^, the fraction of deformed grains decreased to only 12.3%, while the recrystallized fraction increased significantly to 55.9%. At this stage, fine recrystallized grains gradually replaced the deformed structure, resulting in a reduction in the average grain size to 4.38 μm. This indicates that 1.55–1.60 A·mm^−2^ represents the sensitivity window for the rapid microstructural transition of one-pass cold-rolled samples under the present experimental conditions. With further increasing the current density to 1.70 and 1.80 A·mm^−2^, the microstructure was dominated by recrystallized grains, and the recrystallized fraction increased to 57.3% and 82.1%, respectively. Meanwhile, the average grain size decreased to 4.05 and 3.90 μm, respectively. These results demonstrate that increasing the current density effectively promotes the elimination of cold-rolled deformed structures and the formation of recrystallized microstructures.

According to the grain boundary statistics shown in Figure 2, with increasing current density from 1.50 to 1.80 A·mm^−2^, the fraction of LAGBs decreased from 58.9% in the cold-rolled state to 3.5%, while the fraction of HAGBs increased from 41.1% to 96.5%. These results indicate that electrical pulse treatment promotes the transformation of deformation-induced substructures into stable high-angle grain boundary networks associated with recrystallized structures. Meanwhile, the fraction of Σ3^n^ special grain boundaries increased from 8.78% to 30.63%. With the progression of recrystallization, the newly formed high-angle grain boundaries provide favorable sites for the formation of annealing twins, leading to an increased fraction of Σ3^n^ grain boundaries.

These results indicate that variations in current density not only affect the recrystallization state of the material but also regulate grain boundary evolution. At relatively low current densities, recovery is the dominant process. With increasing current density, the material enters a stage of rapid recrystallization. Further increasing the current density promotes the growth and stabilization of recrystallized grains as well as the formation of special grain boundaries. Therefore, controlling the current density enables the microstructural regulation of stainless steel ultra-thin strips from recovery, through partial recrystallization, to a highly recrystallized state [15,16].

#### 3.1.2. Effect of Treatment Duration on Recrystallization Behavior

**Figure 3:**
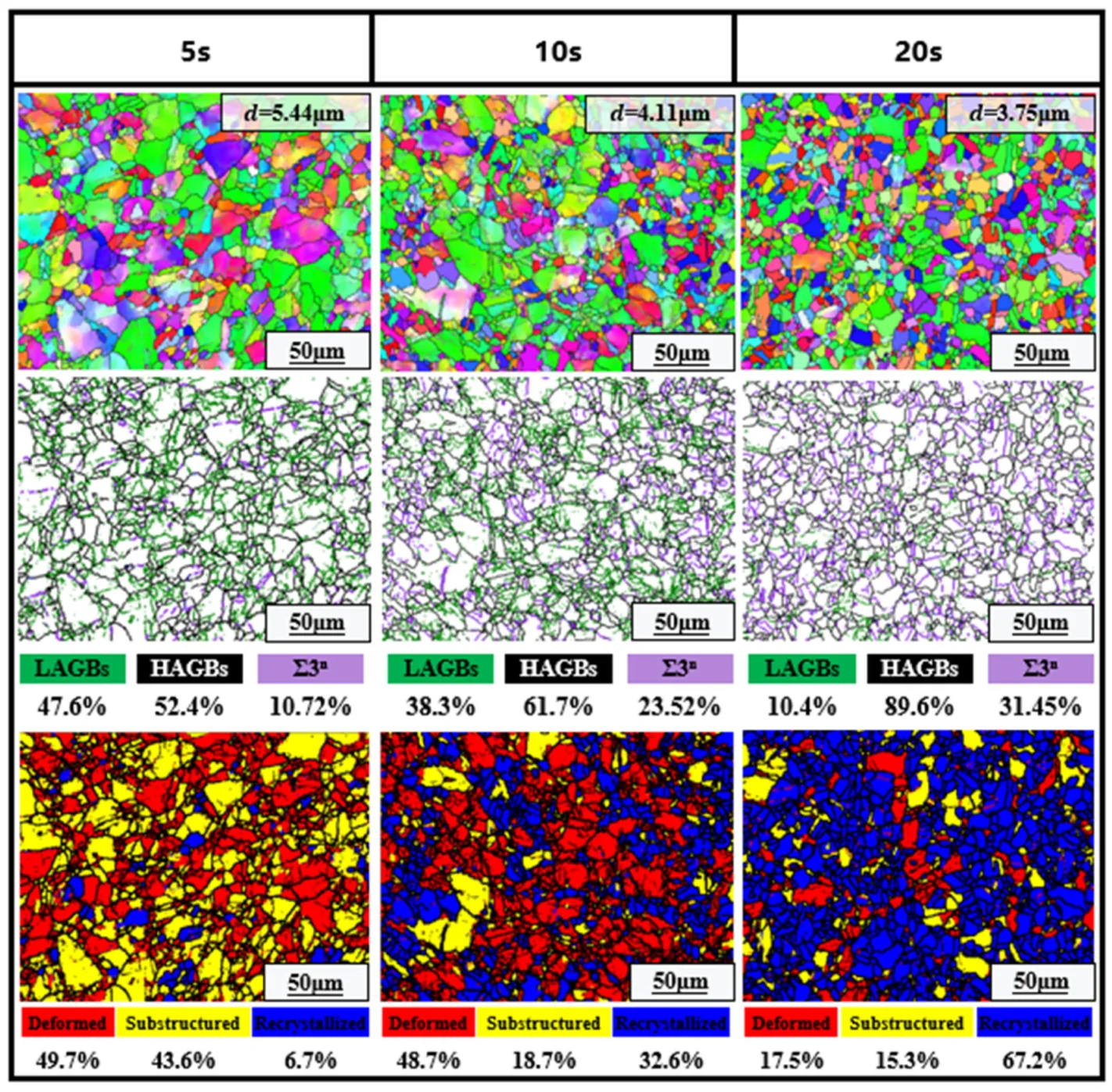
Microstructures of one-pass cold-rolled SUS304 stainless steel ultra-thin strips after electrical pulse treatment with different treatment durations.

Based on a current density of 1.55 A·mm^−2^, a pulse frequency of 100 Hz, and a duty cycle of 10%, electrical pulse treatments with different treatment durations (5, 10, and 20 s) were performed on one-pass cold-rolled samples, and the results are shown in Figure 3. When the treatment duration was 5 s, the electrical pulse-treated samples were mainly composed of deformed grains and substructured grains, with fractions of 49.7% and 43.6%, respectively. When the treatment duration was extended to 10 s, the recrystallized fraction increased to 32.6%, indicating that prolonged electrical pulse treatment promotes the transition from deformed structures to recrystallized structures. With further increasing the treatment duration to 20 s, the recrystallized fraction reached 67.2%, while the fraction of deformed grains decreased to 17.5%. This indicates that continuous electrical pulse treatment facilitates the formation of recrystallized grains, which gradually replace the original deformed structures.

According to the grain boundary statistics shown in Figure 3, when the treatment duration was extended from 5 to 20 s, the fraction of LAGBs decreased from 47.6% to 10.4%, while the fraction of HAGBs increased from 52.4% to 89.6%. Meanwhile, the fraction of Σ3^n^ special grain boundaries increased from 10.72% to 31.45%. These results indicate that increasing the electrical pulse treatment duration promotes the transformation of low-angle boundaries in the original deformed structures into high-angle grain boundaries during recrystallization.

Overall, the electrical pulse treatment duration plays an important role in determining the extent of recrystallization. Prolonging the treatment duration increases the number of pulse cycles and total energy input, providing sufficient time for dislocation movement, subgrain evolution, and grain boundary migration during recrystallization [9,17]. Therefore, controlling the electrical pulse treatment duration enables the regulation of static recrystallization degree and grain boundary characteristics of stainless steel ultra-thin strips.

#### 3.1.3. Effect of Pulse Frequency on Recrystallization Behavior

**Figure 4:**
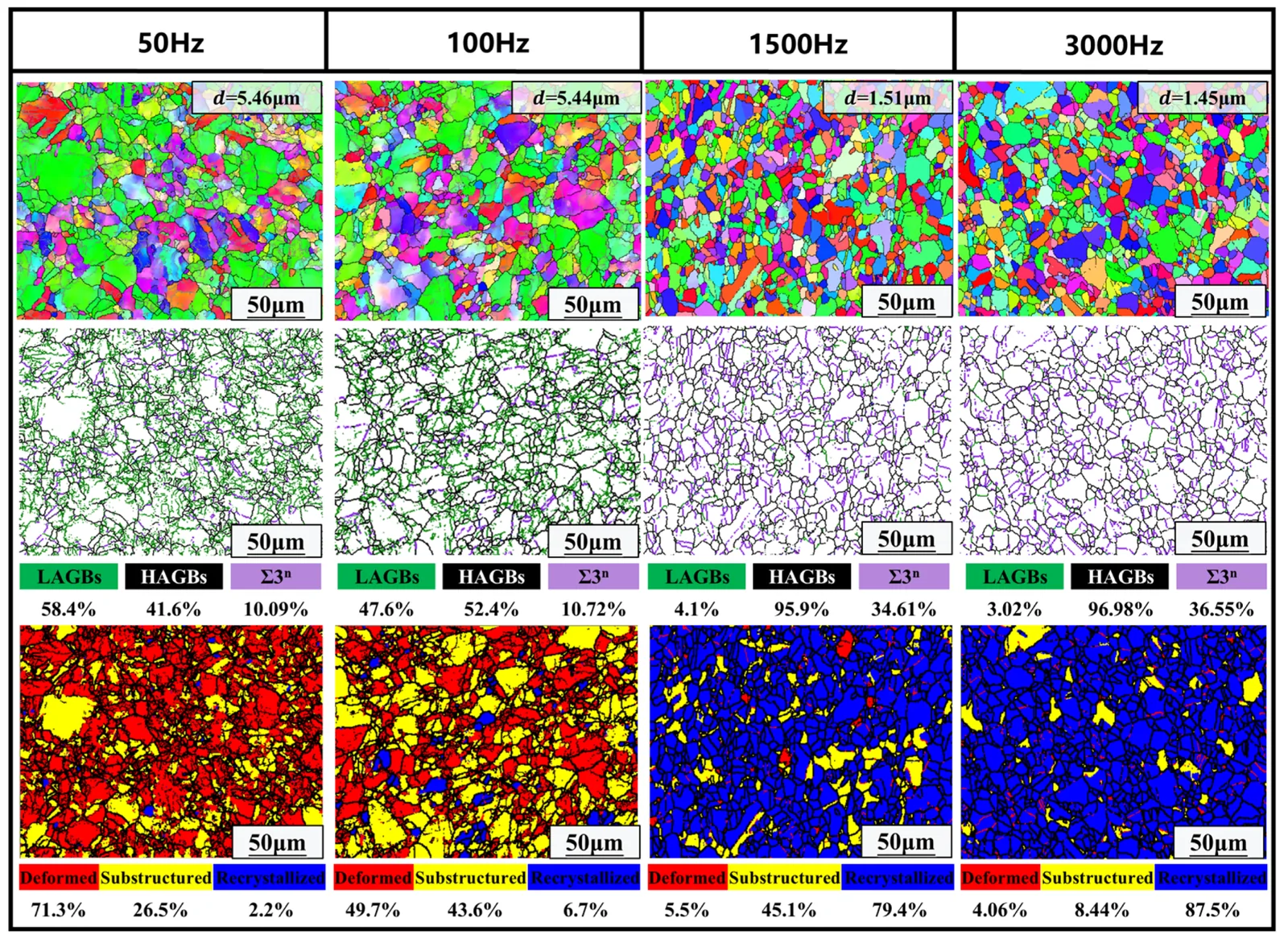
Microstructures of one-pass cold-rolled samples after electrical pulse treatment with different pulse frequencies.

Based on a current density of 1.55 A·mm^−2^, a treatment duration of 5 s, and a duty cycle of 10%, electrical pulse treatments with different pulse frequencies (50, 100, 1500, and 3000 Hz) were performed on one-pass cold-rolled samples. The pulse frequencies were selected as a non-equidistant screening series to compare the recrystallization response across representative low- and high-frequency regimes. The 50 and 100 Hz conditions were used to characterize the low-frequency response, whereas 1500 and 3000 Hz were selected to evaluate the response in the high-frequency regime and to determine whether the effect of further increasing frequency became less significant. The results are shown in Figure 4. At a pulse frequency of 50 Hz, the electrical pulse-treated sample was mainly composed of deformed grains, accounting for approximately 71.3%. When the pulse frequency increased to 100 Hz, localized recovery and recrystallization occurred, with the recrystallized fraction reaching approximately 6.7%. With further increasing the pulse frequency, significant recrystallization occurred in the samples. At 1500 Hz, the recrystallized fraction rapidly increased to 79.4%, while the average grain size decreased to 1.51 μm. When the pulse frequency was further increased to 3000 Hz, the increase in recrystallized fraction became limited, reaching 87.5%, and the average grain size was 1.45 μm. These results indicate that a high degree of recrystallization had already been achieved at this stage, and the effect of further increasing the pulse frequency became less significant.

According to the comparison shown in Figure 4, the evolution of grain boundary characteristics exhibits a strong correspondence with the recrystallization state. When the pulse frequency increased from 50 to 3000 Hz, the fraction of LAGBs decreased from 58.4% to 3.02%, while the fraction of HAGBs increased from 41.6% to 96.98%. This indicates that with increasing recrystallization degree, LAGBs are gradually transformed into HAGBs. Meanwhile, the fraction of Σ3^n^ special grain boundaries increased from 10.09% to 36.55%, suggesting that higher pulse frequencies promote more complete formation of recrystallized grains and grain boundary migration, thereby facilitating the development of special grain boundary networks.

In general, the pulse frequency exhibits a clear frequency-dependent sensitivity during the recrystallization process of stainless steel ultra-thin strips. At 50–100 Hz, the samples remain in the initial stages of recovery and recrystallization. Increasing the pulse frequency from 100 to 1500 Hz significantly enhances the recrystallization degree, accompanied by a synchronous increase in the fractions of HAGBs and Σ3^n^ special grain boundaries. With a further increase to 3000 Hz, the microstructure gradually approaches a fully recrystallized state, while the improvement becomes less significant.

#### 3.1.4. Effect of Duty Cycle on Recrystallization Behavior

**Figure 5:**
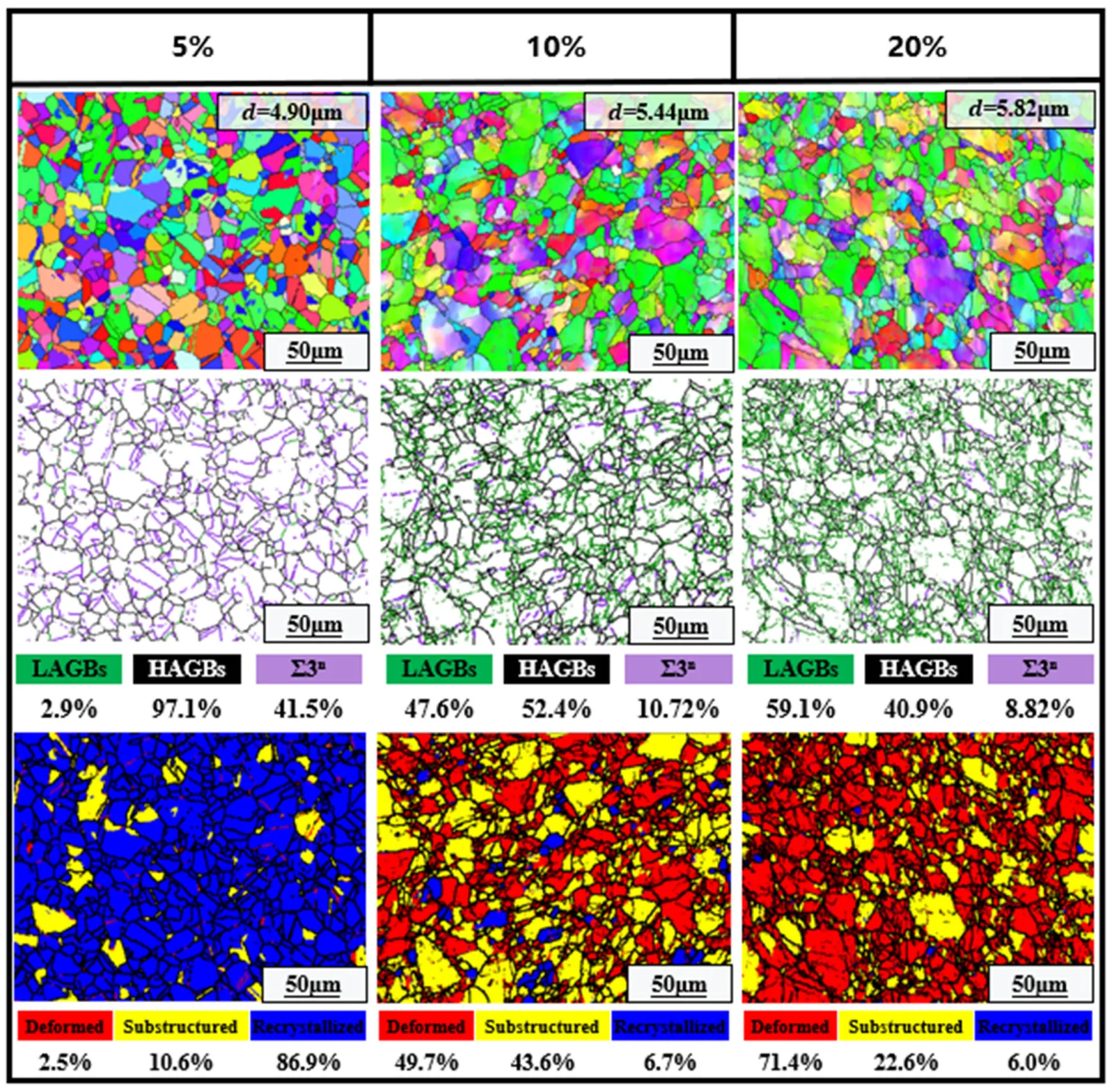
Microstructures of one-pass cold-rolled SUS304 stainless steel ultra-thin strips after electrical pulse treatment with different duty cycles.

The microstructural evolution of one-pass cold-rolled samples was investigated by varying the pulse duty cycle (5%, 10%, and 20%) while maintaining an average current of 2.4 A, a treatment duration of 5 s, and a pulse frequency of 100 Hz. Unlike the current-density-controlled experiments described in Section 3.1.1, Section 3.1.2 and Section 3.1.3, the duty-cycle experiment was conducted under a constant average-current condition. Thus, 2.4 A represents the average current rather than the current density. Under a rectangular pulse waveform, decreasing the duty cycle at constant average current increases the instantaneous pulse current and current density during the pulse-on period, thereby allowing the effect of duty cycle on recrystallization behavior to be evaluated. The results are shown in Figure 5. At a duty cycle of 20%, the electrical pulse-treated sample was mainly composed of deformed grains, accounting for 71.4%. When the duty cycle was decreased to 10%, significant recovery occurred, and the fraction of deformed grains decreased to 49.7%. However, no obvious recrystallization transformation was observed at this stage. With a further decrease in duty cycle to 5%, the recrystallized fraction rapidly increased to 86.9%. At this condition, a large proportion of the original deformed structures transformed into recrystallized grains, resulting in significant microstructural evolution.

According to the comparison shown in Figure 5, the grain boundary characteristic evolution exhibits a trend consistent with the recrystallization behavior. When the duty cycle decreased from 20% to 5%, the fraction of LAGBs decreased from 59.1% to 2.9%, while the fraction of HAGBs increased from 40.9% to 97.1%. This indicates that the rapid increase in recrystallization degree is accompanied by the gradual transformation of deformation-induced substructures into recrystallized grain boundary networks. Meanwhile, the fraction of Σ3^n^ special grain boundaries increased from 8.82% to 41.5%, suggesting that grain boundary migration and reconstruction become more pronounced at higher recrystallization degrees, resulting in a significant increase in special grain boundaries.

Overall, under the condition of constant average current, reducing the duty cycle concentrates the current input within a shorter pulse-on duration, thereby increasing the instantaneous peak current and current density [18]. Olevsky et al. [19] found that both pulse current density and duty cycle exhibit strong parameter sensitivity, and their variations can significantly influence current-induced microstructural responses. High-intensity short-duration electrical pulses can further accelerate the reconstruction of grain boundary and intragranular structures [20,21]. Therefore, under a constant average current, the duty cycle does not simply alter the proportion of current-on time; instead, it significantly affects static recrystallization behavior and grain boundary evolution by regulating the instantaneous intensity of pulse current.

### 3.2. Effect of Rolling Reduction on Recrystallization Behavior of Ultra-Thin Strips After Electrical Pulse Treatment

#### 3.2.1. Relationship Between Rolling Reduction and Stored Dislocations

**Figure 6:**
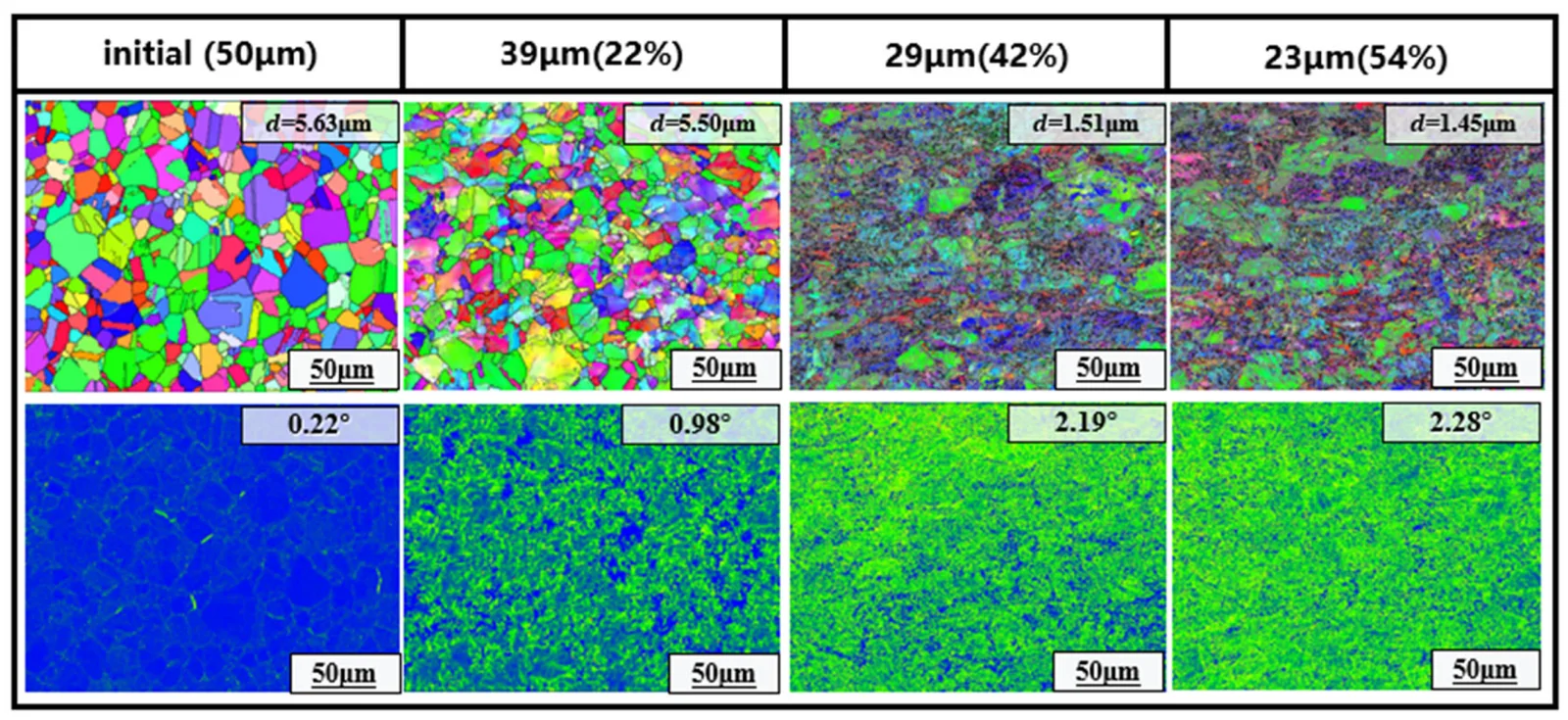
Microstructures of SUS304 stainless steel ultra-thin strips with different rolling reductions.

Figure 6 shows the microstructures of SUS304 stainless steel ultra-thin strips with different rolling reductions. The initial material consisted of uniformly distributed equiaxed grains, with an average grain size of 5.63 μm and an average KAM value of 0.22°. After multi-pass rolling deformation using the 20-high rolling mill, the grains were gradually elongated and fragmented along the rolling direction. With increasing reduction from 22% to 54%, the average grain size decreased from 5.50 μm to 1.45 μm, while the corresponding average KAM values increased to 0.98° and 2.28°, respectively. These results indicate that rolling deformation promotes the development of intragranular orientation gradients and the formation of deformation structures, resulting in increased local misorientation and plastic deformation heterogeneity. Previous studies have also demonstrated that increasing rolling reduction significantly enhances the formation of shear bands, lattice rotation, and local orientation gradients within grains [22,23].

According to the average KAM value, the GND density can be estimated:

(1) $$\rho_{\mathrm{GND}}\approx \frac{2{\mathrm{KAM}}_{\mathrm{ave}}}{\mathrm{\mu b}}$$

where *μ* is the step size, *b* is the Burgers vector [24].

The GND densities under one-pass, three-pass, and five-pass rolling conditions were calculated as approximately 6.71 × 10^14^ m^−2^, 1.50 × 10^15^ m^−2^, and 1.56 × 10^15^ m^−2^, respectively. It can be observed that increasing the rolling reduction significantly promotes the accumulation of GNDs within grains. Notably, when the reduction increased from 42% to 54%, the increments in KAM values, LAGB fractions, and GND density became significantly smaller. This indicates that, at high deformation levels, a dynamic balance is gradually established between dislocation multiplication and dislocation rearrangement/annihilation processes, resulting in a slower rate of dislocation accumulation [25].

#### 3.2.2. Effect of Stored Dislocations on Recrystallization Degree

**Figure 7:**
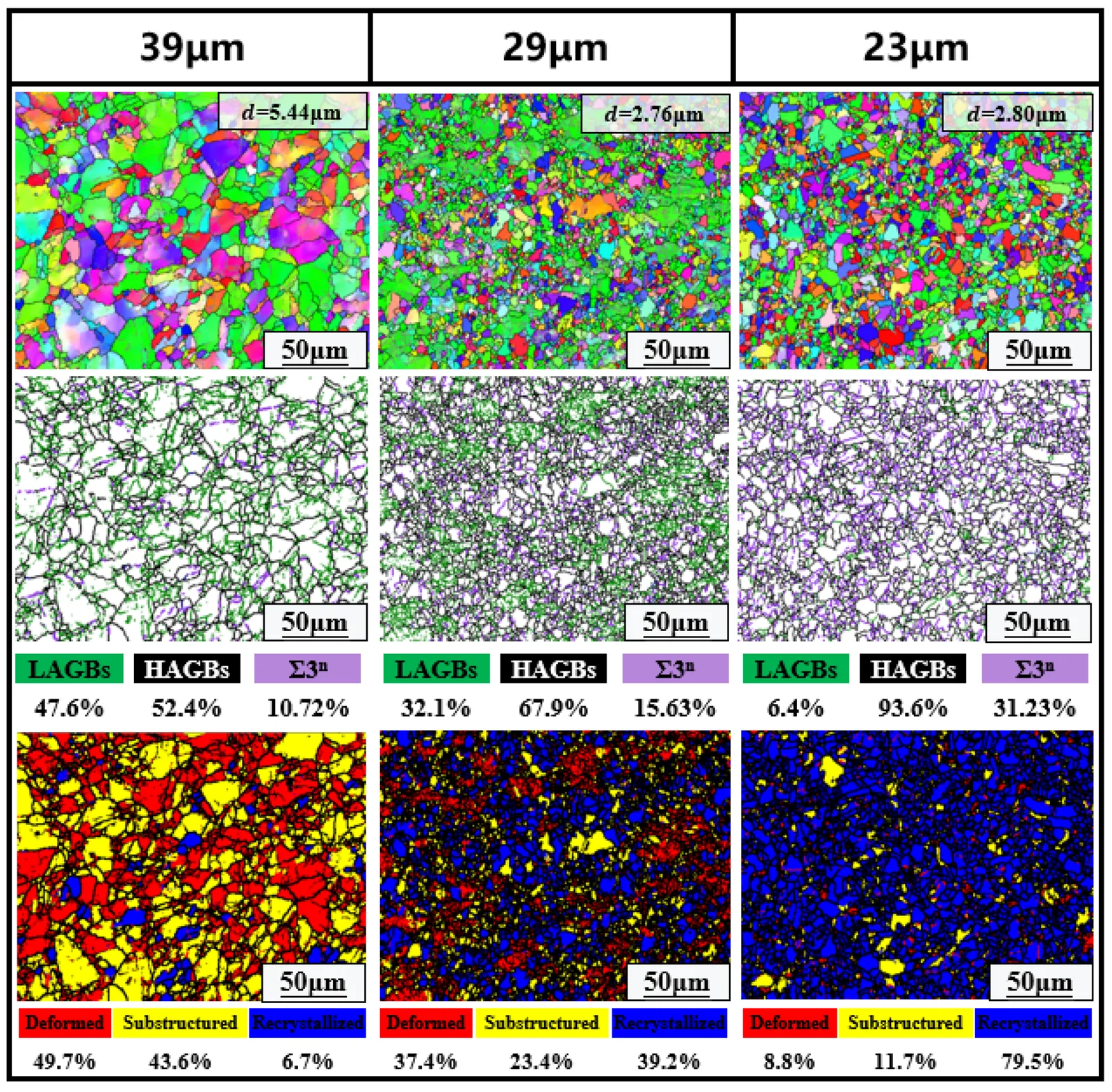
Microstructural morphologies of SUS304 stainless steel ultra-thin strips with different rolling reductions after identical electrical pulse treatment.

Based on the comparison of microstructures under different current densities in Figure 2, it can be observed that the one-pass cold-rolled sample treated under an electrical pulse condition of 100 Hz frequency, 10% duty cycle, 5 s treatment duration, and 1.55 A·mm^−2^ current density consists of deformed grains, substructured grains, and a small fraction of recrystallized grains. This indicates that the sample is at a critical transition stage from recovery to recrystallization. Therefore, this electrical pulse treatment condition was selected to process cold-rolled samples with different reductions, in order to investigate the effect of initial stored dislocations on electrical pulse-induced recrystallization behavior. The corresponding microstructural morphologies are shown in Figure 7.

Cold-rolled samples with different reductions exhibited distinct microstructural evolution under identical electrical pulse treatment conditions. For the sample with a thickness of 39 μm (22% reduction), a large fraction of deformed grains (49.7%) and substructured grains (43.6%) remained after electrical pulse treatment, while the recrystallized fraction was only 6.7%, with an average grain size of 5.44 μm. When the thickness decreased to 29 μm (42% reduction), the fraction of deformed grains decreased to 37.4%, accompanied by the formation of numerous fine equiaxed recrystallized grains. The recrystallized fraction increased to 39.2%, and the average grain size decreased to 2.76 μm. For the 23 μm sample (54% reduction), recrystallized grains became dominant after electrical pulse treatment, with a recrystallized fraction of 79.5% and a deformed grain fraction of only 8.8%. Overall, the average grain size exhibited a decreasing trend with increasing rolling reduction. The average grain size of the 23 μm sample was slightly larger than that of the 29 μm sample. This may be attributed to the higher fraction of deformed grains retained in the 29 μm sample, where the grains had not fully recovered from the fragmented deformation state, resulting in a smaller average grain size.

According to Figure 7, the evolution of grain boundary characteristics exhibits a similar trend to the recrystallization behavior. With increasing rolling reduction, the LAGBs after electrical pulse treatment are gradually replaced by recrystallization-induced HAGBs, resulting in the formation of a more complete grain boundary network. Meanwhile, the fraction of Σ3^n^ special grain boundaries increases monotonically from 10.72% in the 39 μm sample to 31.23% in the 23 μm sample. These results indicate that samples with higher initial dislocation densities exhibit enhanced dislocation rearrangement and stronger recrystallization responses under identical electrical pulse treatment conditions. This difference is mainly associated with the stored energy introduced by cold deformation [26]. Higher rolling reductions generate higher densities of dislocations, deformation bands, and non-equilibrium grain boundaries, thereby increasing the stored deformation energy and providing a greater thermodynamic driving force for subsequent static recrystallization. Meanwhile, these highly defective regions can serve as potential nucleation sites for recrystallized grains [27,28]. Therefore, the 29 μm and 23 μm samples, with higher initial dislocation densities, larger recrystallization driving forces, and more nucleation sites, can achieve faster dislocation consumption and microstructural reconstruction under the same electrical pulse treatment conditions. Taken together, the above results indicate that the recrystallization response under electrical pulse treatment is governed by the combined effects of the applied electrical parameters and the initial deformation state. Increasing current density or treatment duration increases the effective electrical energy input, while variations in pulse frequency and duty cycle alter the temporal characteristics and instantaneous intensity of the electrical loading. At the same time, a higher initial GND density provides a larger stored-energy driving force and a greater number of potential recrystallization nucleation sites. Therefore, the final recrystallized fraction is not determined by a single electrical parameter, but by the coupling between electrical pulse conditions and the initial microstructural state. This coupled dependence provides the physical basis for incorporating both electrical pulse parameters and initial GND density into the quantitative recrystallization model established in Section 3.3.

### 3.3. Regulation of Recrystallization Behavior and Grain Boundary Characteristics by Electrical Pulse Treatment

#### 3.3.1. Constitutive Model for Static Recrystallization

The above results demonstrate that both electrical pulse treatment parameters and the initial microstructural state significantly influence the recrystallization behavior of the material. Based on the classical JMAK recrystallization kinetic model, an electrical pulse treatment-based recrystallization constitutive model was established by incorporating electrical pulse parameters and initial microstructural parameters [29]. In the classical JMAK model, *X* represents the recrystallized fraction, *k* is the recrystallization kinetic rate constant, and *n* is the Avrami exponent.

*X* = 1 − exp (−*kt^n^*) (2)

According to Figure 2, Figure 3, Figure 4, Figure 5, Figure 6 and Figure 7, the recrystallization behavior of cold-rolled stainless steel ultra-thin strips during electrical pulse treatment is governed by the coupled effects of electrical pulse parameters and the initial microstructural state, including current density, pulse frequency, duty cycle, treatment duration, and initial geometrically necessary dislocation density. Based on these key parameters, a recrystallization kinetic model was developed in this study, as presented below.

(3) $$X_{\mathrm{rex}}=X_{0}+\left(1-X_{0}\right)\left(1-\exp\left[-K_{0}{\left(\frac{I}{\mathrm{Dwh}}\right)}^{a}f^{b}\rho_{\mathrm{GND}}^{c}t^{n}\right]\right)$$

where *X_rex_* represents the recrystallized fraction after electrical pulse treatment, *X*_0_ represents the initial recrystallized fraction of the corresponding cold-rolled state, *I* is the average current, *D* is the duty cycle, *w* and *h* represent the width and thickness of the sample, respectively, *f* is the pulse frequency, *ρ_GND_* represents the initial geometrically necessary dislocation density of the sample before electrical pulse treatment, and *t* is the electrical pulse treatment duration. *K*_0_, *a*, *b*, *c*, and *n* are the fitting parameters of the model.

The experimental data were nonlinearly fitted using 1stOpt software (version 5.0), and the fitting parameters *K*_0_ = 1.61, *a* = 17.87, *b* = 0.79, *c* = −2.88, and *n* = 1.34 were obtained. The coefficient of determination of the model was R^2^ = 0.9545, indicating that the proposed model can effectively describe the effects of electrical pulse treatment parameters and the initial rolling state on the static recrystallization fraction within the investigated parameter range.

It should be emphasized that the proposed model is semi-empirical and its present coefficients are calibrated specifically for SUS304 stainless steel ultra-thin strips within the investigated ranges of electrical pulse parameters and initial deformation states. Therefore, extrapolation beyond the present processing window should be performed with caution. For other steel grades, differences in material-dependent properties and recrystallization kinetics may alter the response to electrical pulse treatment, and the model parameters would require recalibration using corresponding experimental data. Thus, the present model should be regarded as a quantitative framework for describing the coupled effects of electrical pulse parameters and initial deformation state rather than as a universal constitutive relationship.

#### 3.3.2. Quantitative Relationship Between Recrystallized Fraction and Grain Boundary Characteristics

As the recrystallization process proceeds during electrical pulse treatment, the cold-rolling-induced deformed substructures are gradually consumed by recrystallized grains, accompanied by a decrease in low-angle grain boundaries and an increase in high-angle grain boundaries [30]. To quantitatively describe the relationship between recrystallization degree and grain boundary characteristics, a correlation model between the HAGB fraction and recrystallized fraction was established by using the recrystallized volume fraction as an intermediate microstructural variable.

(4) $$F_{\mathrm{HAGB}}=F_{\mathrm{HAGB}0}+AX_{\mathrm{rex}}^{m}$$

*F_LAGB_* = 1 − *F_HAGB_* (5)

In the equation, *F_HAGB_* and *F_LAGB_* represent the fractions of high-angle grain boundaries and low-angle grain boundaries, respectively. *F_HAGB_*_0_ represents the initial fraction of high-angle grain boundaries in the cold-rolled state, while *A* and *m* are fitting parameters.

The fitting parameters were obtained from the experimental data *A* = 64.51, *m* = 0.75 and the coefficient of determination was R^2^ = 0.9664. This model is mainly used to describe the overall quantitative relationship between the fraction of Σ3^n^ special grain boundaries and the recrystallized fraction within the experimental parameter range. The high coefficient of determination indicates a strong quantitative correlation between the recrystallized volume fraction and the HAGB fraction.

#### 3.3.3. Correlation Between Recrystallized Fraction and Σ3^n^ Special Grain Boundaries

During electrical pulse treatment, not only do the high-angle grain boundaries and low-angle grain boundaries undergo significant evolution, but the Σ3 boundaries and their related special grain boundaries also experience substantial reconstruction [31,32]. To further describe the relationship between special grain boundaries and the recrystallization process, a correlation model between the fraction of Σ3^n^ special grain boundaries and the recrystallized volume fraction was established.

(6) $$F_{\sum 3^{n}}=F_{\sum 3_{0}^{n}}+BX_{\mathrm{xre}}^{p}{\left(\frac{d}{d_{0}}\right)}^{q}$$

In the equation, $F_{\sum 3^{n}}$ represents the fraction of Σ3^n^ special grain boundaries, $F_{\sum 3_{0}^{n}}$ represents the initial fraction of Σ3^n^ special grain boundaries in the cold-rolled state, and *B*, *p*, and *q* are fitting parameters. The fitting results yielded the values of *B* = 40.15, *p* = 1.21, and *q* = 0.79, with a coefficient of determination of R^2^ = 0.9031. This model is mainly used to describe the overall trend of Σ3^n^ special grain boundary evolution with increasing recrystallization fraction within the experimental range.

Based on the above models, a quantitative relationship framework from the initial rolling state and electrical pulse processing parameters to recrystallized microstructures and grain boundary characteristics can be established. Within the investigated processing window, this framework provides a basis for the inverse estimation of electrical pulse parameters required to achieve a target microstructural state. Given the initial cold-rolled state and the desired recrystallized fraction, the appropriate range of electrical pulse parameters can be obtained through inverse calculation using the recrystallization prediction model. The corresponding fractions of high-angle grain boundaries and special grain boundaries can then be predicted based on the established grain boundary correlation models. Therefore, the qualitative relationships between electrical pulse parameters and microstructural evolution can be further transformed into a regulation pathway of “process parameter input-quantitative microstructure prediction-inverse design of target microstructures”. This provides theoretical and technological guidance for the targeted control of static recrystallization degree and grain boundary characteristics in SUS304 stainless steel ultra-thin strips.

## 4. Conclusions

(1) The electrical pulse parameters exhibit distinct sensitivity windows for the static recrystallization of SUS304 stainless steel ultra-thin strips. With increasing current density and treatment time, the recrystallized fraction continuously increases, and 1.55–1.60 A·mm^−2^ is identified as the sensitive range for current density. When the pulse frequency increases from 50 Hz to 1500 Hz, the recrystallized fraction increases sharply from 2.2% to 79.4%. Meanwhile, reducing the duty cycle from 10% to 5% results in a significant increase in the recrystallized fraction from 6.7% to 86.9%. With increasing recrystallization fraction, the proportion of LAGBs decreases significantly, whereas the proportions of HAGBs and Σ3^n^ special grain boundaries show an overall increasing trend.

(2) Cold-rolling deformation also affects the recrystallized fraction of ultra-thin strips subjected to electrical pulse treatment. When the reduction increases from 22% to 54%, the initial GND density increases from 6.71 × 10^14^ m^−2^ to 1.56 × 10^15^ m^−2^. Under identical electrical pulse treatment conditions, the recrystallized fractions of the samples are 6.7% and 79.5%, respectively, while the GND densities after treatment decrease by approximately 17.4% and 86.0% compared with their corresponding cold-rolled states. These results indicate that electrical pulse treatment induces more significant microstructural evolution in samples with higher stored dislocation densities.

(3) Based on the JMAK model, a semi-empirical prediction model for electrical pulse-assisted static recrystallization was established by introducing variables including current, duty cycle, pulse frequency, treatment time, and initial GND density. The model achieved a high coefficient of determination (R^2^ = 0.9545). Furthermore, quantitative correlations between the recrystallized fraction and the fractions of HAGBs and Σ3^n^ special grain boundaries were established, forming a quantitative relationship among the initial rolling state, electrical pulse processing parameters, recrystallized fraction, and grain boundary characteristics.

## Figures and Tables

**Table 1:** Chemical composition of SUS304 stainless steel ultra-thin strip (%, mass fraction).

C	Cr	Ni	Si	Mn	S	P	Fe
0.06	17.35	8.55	0.42	1.02	0.03	0.05	Bal.

## Data Availability

The original contributions presented in this study are included in the article. Further inquiries can be directed to the corresponding author.
